# Pharmacokinetics of polatuzumab vedotin in combination with R/G-CHP in patients with B-cell non-Hodgkin lymphoma

**DOI:** 10.1007/s00280-020-04054-8

**Published:** 2020-03-28

**Authors:** Colby S. Shemesh, Priya Agarwal, Tong Lu, Calvin Lee, Randall C. Dere, Xiaobin Li, Chunze Li, Jin Y. Jin, Sandhya Girish, Dale Miles, Dan Lu

**Affiliations:** 1 Department of Clinical Pharmacology Oncology, Genentech Inc., 1 DNA Way, South San Francisco, CA 94080 USA; 2 Clinical Science, Genentech Inc., 1 DNA Way, South San Francisco, CA 94080 USA; 3 Bioanalytical Science, Genentech Inc., 1 DNA Way, South San Francisco, CA 94080 USA

**Keywords:** B-cell non-Hodgkin lymphoma, Polatuzumab vedotin, Pharmacokinetics, Combination therapy, Drug interactions, Phase Ib/II

## Abstract

**Purpose:**

The phase Ib/II open-label study (NCT01992653) evaluated the antibody-drug conjugate polatuzumab vedotin (pola) plus rituximab/obinutuzumab, cyclophosphamide, doxorubicin, and prednisone (R/G-CHP) as first-line therapy for B-cell non-Hodgkin lymphoma (B-NHL). We report the pharmacokinetics (PK) and drug–drug interaction (DDI) for pola.

**Methods:**

Six or eight cycles of pola 1.0–1.8 mg/kg were administered intravenously every 3 weeks (q3w) with R/G-CHP. Exposures of pola [including antibody-conjugated monomethyl auristatin E (acMMAE) and unconjugated MMAE] and R/G-CHP were assessed by non-compartmental analysis and/or descriptive statistics with cross-cycle comparisons to cycle 1 and/or after multiple cycles. Pola was evaluated as a potential victim and perpetrator of a PK drug–drug interaction with R/G-CHP. Population PK (popPK) analysis assessed the impact of prior treatment status (naïve vs. relapsed/refractory) on pola PK.

**Results:**

Pola PK was similar between treatment arms and independent of line of therapy. Pola PK was dose proportional from 1.0 to 1.8 mg/kg with R/G-CHP. Geometric mean volume of distribution and clearance of acMMAE ranged from 57.3 to 95.6 mL/kg and 12.7 to 18.2 mL/kg/day, respectively. acMMAE exhibited multi-exponential decay (elimination half-life ~ 1 week). Unconjugated MMAE exhibited formation rate-limited kinetics. Exposures of pola with R/G-CHP were similar to those in the absence of CHP; exposures of R/G-CHP in the presence of pola were comparable to those in the absence of pola.

**Conclusions:**

Pola PK was well characterized with no clinically meaningful DDIs with R/G-CHP. Findings are consistent with previous studies of pola + R/G, and support pola + R/G-CHP use in previously untreated diffuse large B-cell lymphoma.

**Electronic supplementary material:**

The online version of this article (10.1007/s00280-020-04054-8) contains supplementary material, which is available to authorized users.

## Introduction

B-cell non-Hodgkin lymphoma (B-NHL) is a heterogeneous set of malignancies with varying clinical outcomes. The outcomes of B-NHL vary depending on many factors including histology; while certain patients can be cured of disease, others remain refractory or relapse. The current standard of care for the majority of patients with B-NHL is immunochemotherapy; however, treatment-related morbidity and lack of efficacy remain an obstacle, especially for middle aged/older and/or frail patients [1, 2]. As such, a substantial unmet medical need in patients with NHL remains, requiring evaluation of alternate and more effective approaches [3, 4].

Antibody-drug conjugates (ADCs) have become a rapidly emerging class of therapeutics, aiming to revolutionize the field of cancer therapy including the treatment of B-NHL [5, 6]. ADCs are composed of a monoclonal antibody (mAb) bound to a potent cytotoxic agent through a linker to provide targeted delivery to tumor cells. Attempts to treat B-cell malignancies with ADCs in a targeted setting offer several advantages over traditional chemotherapy with enhanced biologic activity [7]. Across both hematologic and solid tumors, five ADCs have been approved including: gemtuzumab ozogamicin (Mylotarg™), brentuximab vedotin (Adcetris™), ado-trastuzumab emtansine (Kadcyla™), inotuzumab ozogamicin (Besponsa™) and polatuzumab vedotin (pola; Polivy™). Over 65 additional ADCs are currently in clinical evaluation targeting a wide variety of tumors [8–10].

One broad and attractive targeting option for B-NHL using an ADC is the CD79 signaling component of the B-cell receptor, due to its exclusive expression on the B-cell lineage and malignant B cells in patients with B-NHLs [11]. Pola was recently approved by the United States Food and Drug Administration (US FDA) in combination with bendamustine and rituximab for patients with relapsed/refractory (R/R) diffuse large B-cell lymphoma (DLBCL) after at least two prior therapies [12]. Pola is comprised of a CD79b-specific humanized monoclonal antibody that is conjugated to the cytotoxic agent monomethyl auristatin E (MMAE) via a protease-cleavable peptide linker. Pola selectively binds to the CD79b portion of the B-cell receptor present on the surface of malignant and non-malignant B cells, triggering internalization of the complex by the cell. After internalization, the linker is cleaved, resulting in the intracellular release of MMAE, which binds to tubulin to inhibit polymerization triggering tumor cell death [11]. Pola has demonstrated promising activity with combination agents in several clinical trials in patients with B-NHL and continues to be investigated in a phase III clinical trial [13–18]. Combining pola with rituximab or obinutuzumab plus cyclophosphamide, doxorubicin, and prednisone (R/G-CHP) has a manageable safety profile and promising preliminary clinical activity in newly diagnosed DLBCL [19], supporting the initiation of a phase III trial comparing pola + R-CHP to rituximab, cyclophosphamide, doxorubicin, vincristine, and prednisone (R-CHOP).

Given that pola is administered in combination with drugs from an established standard of care, a comprehensive evaluation of clinically relevant drug–drug interactions (DDI) is necessary. Due to the complexity of ADCs, understanding their pharmacokinetics (PK) and DDI can be challenging [20–23]. DDI studies are regularly conducted with small-molecule drugs (SMDs), but few have been evaluated for large molecules such as therapeutic proteins (TPs), mAbs, and/or ADCs, which have complex elimination processes.

Labeling language around DDI for marketed ADCs is uniquely intricate, with findings based on dedicated trials, population PK studies, non-compartmental analysis, visual/graphical inspection of exposure, and/or physiologically based PK modeling approaches. European Medicines Agency and FDA recommendations for clinical pharmacology studies with ADCs are still evolving; however, sponsors are aware of the necessity to evaluate DDI potential [24, 25].

A theoretical DDI could result from a pharmacologic effect of a TP and SMD in combination, via the cell-surface target antigen, immunogenicity modulation (e.g., drugs causing immunosuppression and altering antidrug–antibody production), impacting target-mediated drug disposition (TMDD) and/or CYP450 modulation [26–28]. MMAE (the SMD and payload component of pola), doxorubicin, and cyclophosphamide are substrates of CYP3A4 isoenzymes that exhibit variable activity across patients. Concomitant medications that are inhibitors or inducers of the same metabolic isoenzymes can influence and alter the exposure of active drugs, impacting clinical outcomes and toxicity from associated therapies. An additional theoretical PK drug–disease–drug interaction risk exists with pola, rituximab, and obinutuzumab given that these agents have the potential influence of circulating B-cell count and tumor burdens that may in turn affect TMDD for these agents.

The present article describes the PK of pola in a phase Ib/II open-label study of pola in combination with R/G-CHP. The investigation (1) provides insight on the PK of pola in combination with R/G-CHP, (2) evaluates within-study comparisons of pola PK between first-line B-NHL treatment groups, (3) enables cross-study comparisons of pola PK and rituximab/obinutuzumab PK from similar clinical studies employing the same agents, and lastly (4) assesses potential DDIs involving pola when used in combination with R/G-CHP. The primary objective of these analyses is to support the development of pola combination regimens as a first-line treatment for patients with B-cell NHL.

## Materials and methods

### Study design

This was a phase Ib/II, multicenter, open-label, dose-escalation study (GO29044; NCT01992653). Patients with B-NHL received six or eight cycles of pola 1.0–1.8 mg/kg + R/G-CHP (21-day cycles; R/G-CHP was given as per the standard regimen). Patients were given either six or eight cycles of treatment based on the discretion of the investigator in accordance with local institutional practice. All patients with DLBCL (*N* = 75) and follicular lymphoma (FL, *N* = 3) were treatment naïve, except for one patient with FL who had R/R disease. Additional histologies represented included mantle cell lymphoma (*N* = 2) and marginal zone lymphoma (*N* = 1). The 1.8 mg/kg pola dose was selected for combination with R/G-CHP in an expansion phase for front-line treatment of patients with DLBCL [19]. The details of the treatment groups and study phases are summarized in Online Resource 1.

### Baseline characteristics of patients with DLBCL receiving 1.8 mg/kg of polatuzumab vedotin

A total of 66 patients with DLBCL were treated with the recommended phase 2 dose of 1.8 mg/kg of polatuzumab vedotin with R/G-CHP. Median patient age (interquartile range) was 67.5 (64.0–74.0) years, and 34 patients (52%) were male. Eastern Cooperative Oncology Group performance status was 0–1 in 47 (71%) patients, and 2 in 19 (29%) patients. Fifty-six patients (85%) had stage III–IV disease. International Prognostic Index was 0–1 in 4 (6%) patients, and 2, 3, and 4–5 in 19 (29%), 21 (32%), and 22 (33%) patients, respectively. Further breakdown of baseline characteristics for other B-NHL patients receiving doses of polatuzumab vedotin < 1.8 mg/kg in the study was not provided herein as this has been reported previously [19].

### PK sampling and analytical methods

The PK of pola analytes (including antibody-conjugated MMAE [acMMAE], total antibody, and unconjugated MMAE) were characterized [29, 30], in addition to the PK of rituximab, obinutuzumab, cyclophosphamide, and doxorubicin. Due to the high correlation between total antibody and acMMAE (unpublished data on file), only acMMAE and unconjugated MMAE are reported (Online Resource 1). Validated methods were used to determine plasma/serum concentrations of the analytes of interest. The assays were developed and validated in accordance with industry best practices, and the performance parameters of these assays were within industry and health authority recommendations for biopharmaceutics [31–33].

Plasma concentrations of acMMAE were quantified by immunoaffinity liquid chromatography with tandem mass spectrometry (LC–MS/MS). Pola and ADC internal standard were immobilized onto the Protein A resin and the peptide linker was enzymatically cleaved to release MMAE. The released MMAE was measured by LC–MS/MS. The lower limit of quantitation (LLOQ) for the acMMAE assay was 0.359 ng/mL acMMAE (0.50 nM acMMAE) in human plasma. Unconjugated MMAE plasma concentrations were determined by protein-precipitation extraction with LC–MS/MS. Samples were fortified with internal standard (deuterated MMAE) and analytes were isolated through protein precipitation. The LLOQ for the unconjugated MMAE assay was 0.0359 ng/mL MMAE (0.05 nM MMAE) in human plasma. Rituximab and obinutuzumab serum concentrations were determined via enzyme-linked immunosorbent assays. The LLOQs for the rituximab and obinutuzumab assays were 500 ng/mL and 4.05 ng/mL in human serum, respectively. Plasma concentrations of cyclophosphamide were determined by liquid extraction/LC–MS/MS, and doxorubicin by protein precipitation/LC–MS/MS. The LLOQ for the cyclophosphamide assay was 100 ng/mL in human plasma. Circulating metabolites of cyclophosphamide such as 4-hydroxycyclophosphamide were not assessed. For all assays, quality control samples were included to judge assay acceptability.

### Pharmacokinetic analysis

Non-compartmental analysis (NCA) was computed using pola cycle 1 data. NCA parameters were estimated using Phoenix WinNonlin version 6.4.0.768 (Pharsight, Inc., Mountain View, CA). PK parameters included: terminal half-life (*t*_1/2_; acMMAE only), time to maximum concentration (*T*_max_), maximum concentration (*C*_max_), area under the concentration–time curve from time 0 to the last quantifiable time point (AUC_last_; unconjugated MMAE only), AUC from time 0 to infinity (AUC_inf_; acMMAE only), volume of distribution (*V*_ss_), and clearance (CL; acMMAE only). For each value, the geometric mean and % geometric coefficients of variation (CV%) were calculated.

Pola was descriptively assessed as a ‘perpetrator’ of DDIs with cyclophosphamide and doxorubicin by comparing cyclophosphamide and doxorubicin exposure between cycle 1, day 1 (prior to first pola dose on cycle 1, day 2) and cycle 3, day 1 (after pola dosing); prednisone was not assessed in this analysis given the wide therapeutic window of steroids and low risk for pola as a perpetrator of a PK DDI for prednisone. To evaluate pola as a ‘perpetrator’ for DDIs with rituximab/obinutuzumab, data were compared with those for rituximab/obinutuzumab exposure from historical studies [Study BO22334 (NCT01200758): R-CHOP or rituximab, cyclophosphamide, vincristine sulfate, and prednisone (R-CVP), with intravenous R as first-line treatment for FL; Study BO21003 (NCT00576758): obinutuzumab for R/R indolent B-NHL]. Additionally, pola was assessed as a DDI ‘victim’ of CHP by comparing pola exposure with data from previous studies as a comparator, which included Study GO27834 where pola was administered with R/G in the absence of CHP. Patients in Study GO27834 followed the same pola PK sampling scheme in comparison to the current study. No formal statistical testing was performed.

### Population pharmacokinetic (popPK) analysis to assess PK in treatment-naïve versus R/R NHL patients

A two-analyte integrated popPK model that simultaneously describes concentrations of acMMAE and unconjugated MMAE following repeated administrations of pola was previously developed based on data from four clinical studies of pola in B-NHL patients (*N* = 460) who received either single-agent pola, pola with R/G, pola with R/G and bendamustine, or pola + R/G-CHP [DCS4968g (NCT01290549), GO27834 (NCT01691898), GO29365 (NCT02257567), and GO29044] [34]. A two-compartment model with a nonspecific, time-dependent linear clearance, a linear time-dependent exponentially declining clearance, and a Michaelis–Menten clearance provided a good fit of the acMMAE plasma PK profiles. All three acMMAE elimination pathways contributed to the input to the central compartment of unconjugated MMAE, which was also described by a two-compartment model. Population PK parameters, covariate effects, and interindividual variability of model parameters were estimated. The impact of clinically relevant covariates on PK exposures of each analyte were quantified to support key label claims. The details of model building, validation and covariate assessment process were presented by Lu et al. [34]. The popPK analysis was conducted via nonlinear mixed-effects modeling with Nonlinear Mixed-Effect Modeling (NONMEM) software, version 7.3.0 (ICON Development Solutions, Ellicott City, MD) using the first-order conditional estimation method with eta-epsilon interaction.

The popPK analysis described above was applied to assess the impact of line of therapy (treatment-naïve status vs. R/R status) on pola PK. All treatment-naïve patients (*N* = 80) were from GO29044 (*N* = 82), who were all co-administered CHP, making the impact of CHP combination or line of therapy indistinguishable in the current data. Simulations of cycle 6 exposures [AUC and *C*_max_; pola 1.8 mg/kg every 3 weeks (q3w)] for all patients in the four studies were performed by the final population PK model based on individual empirical Bayes estimates of PK parameters using individual covariate values, except that all patients were assumed to have co-administered anti-CD20 therapy. This approach, referred to as partial covariate correction (pCC), adjusted for the potential impact of R/G combination on pola PK. The exposure estimates of pola, including acMMAE and unconjugated MMAE in patients with R/R NHL receiving pola with R/G (*N* = 380), were compared with those in treatment-naïve patients with B-NHL (*N* = 80) receiving pola + R/G-CHP. Geometric means and CV% were computed and tabulated, as well as the geometric mean ratio to the reference category (R/R) and its 90% confidence interval (CI).

## Results

### PK of pola in patients with B-NHL in the phase Ib dose-escalation arm when given in combination with R/G-CHP

**Fig. 1:**
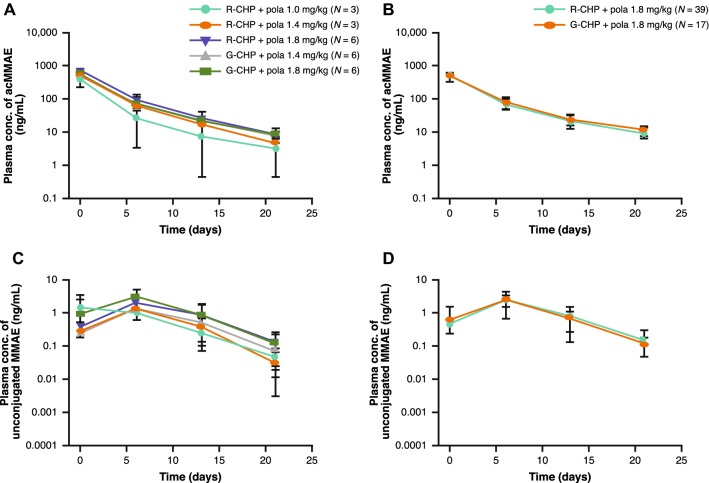
Mean (SD) cycle 1 plasma/serum concentration time profiles of pola by study phase and treatment in patients receiving pola 1.0–1.8 mg/kg in combination with R-CHP, or pola 1.4–1.8 mg/kg in combination with G-CHP. Dose-escalation arms are provided in **a**, **c**, with dose expansion arms in **b**, **d**. Pola analytes include acMMAE (**a**, **b**) and unconjugated MMAE (**c**, **d**). *acMMAE* antibody-conjugated MMAE, *conc* concentration, *G-CHP* obinutuzumab, cyclophosphamide, doxorubicin, and prednisone, *MMAE* monomethyl auristatin E, *pola* polatuzumab vedotin, *R-CHP* rituximab, cyclophosphamide, doxorubicin, and prednisone, *SD* standard deviation

**Fig. 2:**
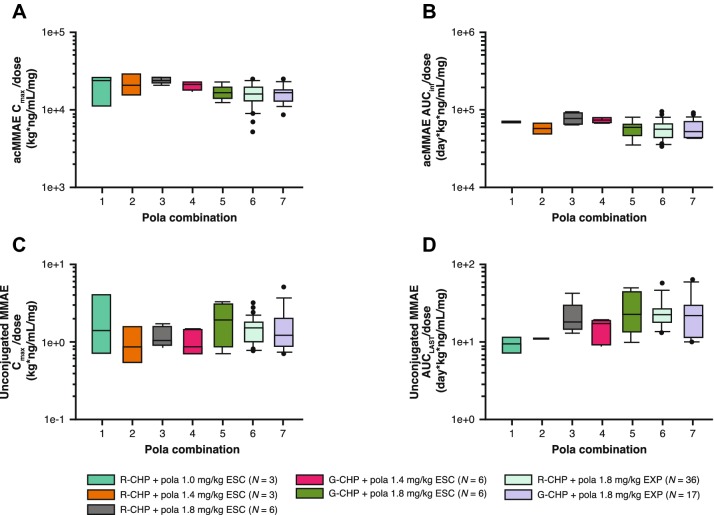
Comparison of dose-normalized exposure of pola by *C*_max_ (**a**, **c**) or AUC (**b**, **d**) within cycle 1 across dose-escalation and expansion arms in patients with B-NHL or DLBCL receiving pola 1.0–1.8 mg/kg + R-CHP or pola 1.4–1.8 mg/kg + G-CHP. Pola analytes include acMMAE (**a**, **b**) and unconjugated MMAE (**c**, **d**). Vertical boxplots include the median, 10th, 25th, 75th, and 90th percentiles as vertical boxes with error bars for each respective cohort and outliers (black bulleted circles) plotted as single point. *acMMAE* antibody-conjugated MMAE, *AUC*_*inf*_ area under the concentration–time curve from 0 to infinity, *AUC*_*last*_ area under the concentration–time curve from 0 until the last measurable time point, *B-NHL* B-cell non-Hodgkin lymphoma, *C*_*max*_ maximum concentration, *DLBCL* diffuse large B-cell lymphoma, *ESC* dose-escalation phase, *EXP* expansion phase, *G-CHP* obinutuzumab, cyclophosphamide, doxorubicin, and prednisone, *MMAE* monomethyl auristatin E, *pola* polatuzumab vedotin, *R-CHP* rituximab, cyclophosphamide, doxorubicin, and prednisone

Non-compartmental analysis and descriptive statistic results, including the mean acMMAE and unconjugated MMAE plasma concentration time profiles and PK parameters across study phase, treatment group, and dose, are provided in Figs. 1a–d and 2a–d, and Table 1. Plasma concentrations of acMMAE reached *C*_max_ at the end of infusion, while plasma concentrations of unconjugated MMAE increased slowly and reached peak values at approximately 6 days. Given the relatively sparse sampling of unconjugated MMAE, the observed *T*_max_ might not reflect the actual *T*_max_ (which is approximately 3 days based on Study DCS4968g [11]).

**Table 1:** Cycle 1 non-compartmental pharmacokinetic parameter summary of pola

Stage/population	Cohort	*N*	*C*_max_ (ng/mL)	AUC_inf_ (ng day/mL)	*t*_1/2_ (day)	*V*_ss_ (mL/kg)	CL (mL/kg/day)
**Conjugate (evaluated as acMMAE)**							
Dose escalation/B-NHL	R-CHP + pola 1.0 mg/kg	3	350 (49.1)	1300 (1.70)^a^	4.99 (18.2)^a^	57.7 (17.3)^a^	14.0 (2.70)^a^
	R-CHP + pola 1.4 mg/kg	3	517 (33.9)	1490 (24.0)^a^	4.82 (15.0)^a^	80.0 (12.5)^a^	17.0 (24.7)^a^
	R-CHP + pola 1.8 mg/kg	6	778 (9.70)	2580 (16.0)	4.75 (14.2)	57.3 (12.6)	12.7 (16.1)
	G-CHP + pola 1.4 mg/kg	5	534 (11.2)	1930 (8.20)	5.18 (8.10)	67.6 (10.9)	13.2 (9.40)
	G-CHP + pola 1.8 mg/kg	6	548 (20.0)	1800 (27.6)	4.87 (10.5)	85.6 (23.5)	18.2 (27.7)
Expansion/DLBCL	R-CHP + pola 1.8 mg/kg	36	503 (36.5)	1800 (28.5)^b^	4.99 (13.2)^b^	91.6 (33.1)^b^	18.2 (28.5)^b^
	G-CHP + pola 1.8 mg/kg	17	513 (26.6)	1890 (23.7)^c^	5.45 (14.2)^c^	95.6 (29.8)^c^	17.3 (23.4)^c^

Stage/population	Cohort	*N*	*C*_max_ (ng/mL)	AUC_last_ (ng day/mL)	*T*_max_ (day)
**Unconjugated MMAE**					
Dose escalation/B-NHL	R-CHP + pola 1.0 mg/kg	3	1.60 (107)	9.22 (33.2)^a^	5.92 (0.08–6.20)
	R-CHP + pola 1.4 mg/kg	3	1.27 (57.2)	10.9^d^	5.95 (5.80–7.08)
	R-CHP + pola 1.8 mg/kg	6	2.11 (29.2)	19.5 (46.6)	5.98 (5.87–11.9)
	G-CHP + pola 1.4 mg/kg	6	1.35 (38.0)	13.4 (38.3)^e^	5.84 (4.86–6.90)
	G-CHP + pola 1.8 mg/kg	6	3.00 (69.7)	22.9 (74.1)^e^	5.85 (0.09–5.94)
Expansion/DLBCL	R-CHP + pola 1.8 mg/kg	35	2.43 (37.9)	22.6 (40.4)^f^	5.90 (2.87–6.99)
	G-CHP + pola 1.8 mg/kg	14	2.44 (60.0)	20.4 (62.8)^g^	5.35 (0.09–6.02)

All values are geometric mean (% geo CV), except for *T*_max_ (median [range])

*acMMAE* antibody-conjugated MMAE, *AUC*_*inf*_ area under the concentration–time curve from 0 to infinity, *AUC*_*last*_ area under the concentration–time curve from 0 until the last measurable time point, *B-NHL* B-cell non-Hodgkin lymphoma, *CL* clearance, *C*_*max*_ maximum concentration, *DLBCL* diffuse large B-cell lymphoma, *G-CHP* obinutuzumab, cyclophosphamide, doxorubicin, and prednisone, *MMAE* monomethyl auristatin E, *pola* polatuzumab vedotin, *R-CHP* rituximab, cyclophosphamide, doxorubicin, and prednisone, *t*_*1/2*_ terminal half-life, *T*_*max*_ time to maximum concentration, *Vss* volume of distribution

^a^*N* = 2; ^b^*N* = 28; ^c^*N* = 11; ^d^*N* = 1; ^e^*N* = 5; ^f^*N* = 27; ^g^*N* = 10

As shown in Table 1, patients with B-NHL receiving pola 1.0–1.8 mg/kg + R-CHP or pola 1.4–1.8 mg/kg + G-CHP had a geometric mean cycle 1 *C*_max_ ranging from 350 to 778 ng/mL (*N* = 3–6) for acMMAE, and 1.27–3.00 ng/mL (*N* = 3–6) for unconjugated MMAE. Increases in the cycle 1 *C*_max_ of acMMAE and unconjugated MMAE were approximately dose proportional (Fig. 2a, c) over the pola dose of 1.0–1.8 mg/kg.

The distribution of acMMAE was mostly restricted to the central compartment, with a geometric mean *V*_ss_ ranging 57.3–85.6 mL/kg (*N* = 2–6) for acMMAE. The CL of acMMAE was low, with a geometric mean ranging 12.7–18.2 mL/day/kg (*N* = 2–6) (Table 1). The concentration–time profile of acMMAE displayed multi-exponential decay, with a geometric mean apparent *t*_1/2_ ranging 4.75–5.18 days (*N* = 2–6) (Table 1). Total systemic exposure by geometric mean ranged 1300–2580 ng day/mL (*N* = 2–6) for acMMAE, and 9.22–22.9 ng day/mL (*N* = 2–6) for unconjugated MMAE across pola + R/G-CHP groups. Similar to observations for *C*_max_, increases in cycle 1 AUC of acMMAE and unconjugated MMAE were generally dose proportional (Table 1, Fig. 2b, d).

The systemic exposure of unconjugated MMAE was low relative to acMMAE. At the 1.8 mg/kg pola dose, geometric mean values for unconjugated MMAE *C*_max_ and AUC were < 2% of those for acMMAE. After three cycles of pola, plasma pre-dose concentrations of acMMAE were 2.00- to 3.71-fold higher than those seen after the first dose, indicative of accumulation with repeated dosing. However, upon repeated administration of pola, pre-dose concentrations of unconjugated MMAE remained low, and no accumulation of unconjugated MMAE was observed with the q3w regimen.

### PK of pola in patients with DLBCL in the phase II expansion arm when given in combination with R/G-CHP

The PK characteristics of pola in patients with DLBCL in the phase II expansion arms receiving pola 1.8 mg/kg + R/G-CHP were generally similar to those in patients with mixed subtype B-NHL in the phase Ib dose-escalation study at the same dose level. They were also similar between the R-CHP and G-CHP treatment groups (Fig. 1a–d).

Across expansion arms in patients receiving pola 1.8 mg/kg + R/G-CHP, the geometric mean cycle 1 *C*_max_ ranged 503–515 ng/mL (*N* = 17–36) for acMMAE, and 2.43–2.44 ng/mL (*N* = 14–35) for unconjugated MMAE, while the geometric mean AUC_inf_ ranged 1800–1890 ng day/mL (*N* = 11–28) for acMMAE, and the geometric mean AUC_last_ was 20.4–22.6 ng day/mL (*N* = 10–27) for unconjugated MMAE (Table 1). Pre-dose concentrations of unconjugated MMAE remained low, with no accumulation of unconjugated MMAE observed with the q3w regimen.

### Potential PK drug–drug interaction of pola and R/G-CHP

The potential for PK DDI between CHP and pola was evaluated using exposure comparisons between patients receiving pola + R/G-CHP and those with R/R DLBCL or FL receiving pola with R/G in a phase II study [17, 35]. Exposure comparisons included cycle 1 *C*_max_ and AUC of each pola analyte including acMMAE and unconjugated MMAE. Pola was not a victim of a drug–drug interaction with CHP as there were no clinically meaningful differences between geometric mean exposures of acMMAE and unconjugated MMAE after the first 1.8-mg/kg dose of pola + R/G-CHP vs. pola + R/G (Table 2), as summarized below. It is worth noting that, as mentioned in the method section for popPK analysis, the effect of CHP combination is indistinguishable from line of therapy (treatment-naïve vs. R/R status).

**Table 2:** Assessment of CHP as a perpetrator of a PK drug–drug interaction with 1.8 mg/kg of pola as a victim based on cycle 1 non-compartmental analysis results

Analyte	Parameter	*N*	GO29044 (pola + R-CHP) DLBCL	*N*	GO27834 (pola + R) FL	GMR (90% CI)
acMMAE	*C*_max_ (ng/mL)	36	503 (36.4)	17	780 (14.4)	0.646 (0.576–0.724)
	AUC_inf_ (ng day/mL)	28	1800 (28.5)	15	2530 (25.9)	0.711 (0.616–0.820)
Unconjugated MMAE	*C*_max_ (ng/mL)	35	2.43 (37.9)	20	1.75 (54.3)	1.39 (1.11–1.73)
	AUC_last_ (ng day/mL)	27	22.6 (40.4)	20	15.8 (50.1)	1.43 (1.15–1.78)

Analyte	Parameter	*N*	GO29044 (pola + G-CHP) DLBCL	*N*	GO27834 (pola + G) DLBCL	GMR (90% CI)
acMMAE	*C*_max_ (ng/mL)	17	513 (26.6)	33	694 (22.7)	0.739 (0.651–0.839)
	AUC_inf_ (ng day/mL)	11	1890 (23.7)	26	2350 (28.4)	0.805 (0.691–0.938)
Unconjugated MMAE	*C*_max_ (ng/mL)	14	2.44 (60.0)	40	2.68 (81.1)	0.911 (0.664–1.25)
	AUC_last_ (ng day/mL)	10	20.4 (62.8)	40	22.5 (70.7)	0.907 (0.629–1.31)

All values are geometric mean (% geo CV), except for GMR

*acMMAE* antibody-conjugated MMAE, *AUC*_*inf*_ area under the concentration–time curve from 0 to infinity, *AUC*_*last*_ area under the concentration–time curve from 0 until the last measurable time point, *CHP* cyclophosphamide, doxorubicin, and prednisone, *CI* confidence interval, *C*_*max*_ maximum concentration, *CV* coefficient of variation, *DLBCL* diffuse large B-cell lymphoma, *FL* follicular lymphoma, *G* obinutuzumab, *G-CHP* obinutuzumab, cyclophosphamide, doxorubicin, and prednisone, *GMR* geometric mean ratio, *MMAE* monomethyl auristatin E, *PK* pharmacokinetic, *pola* polatuzumab vedotin, *R* rituximab, *R-CHP* rituximab, cyclophosphamide, doxorubicin, and prednisone

For exposure assessments of pola + R-CHP compared with pola combined with rituximab (without CHP), a direct comparison in patients of the same B-NHL type was not possible. However, given DLBCL and FL patients have generally similar PK for pola, a cross-study comparison of available data was conducted. Within cycle 1, exposure of pola in patients with treatment-naïve DLBCL receiving pola + R-CHP showed a geometric mean ratio (GMR) for AUC of 0.711 (90% CI 0.616–0.820) for acMMAE and 1.43 (90% CI 1.15–1.78) for unconjugated MMAE when compared with R/R FL patients receiving pola with rituximab (in the absence of CHP); this is likely reflective of cross-study variations and within the variability of each analyte (~ 30% for acMMAE, and ~ 60% for unconjugated MMAE) (Table 2).

For the obinutuzumab-containing cohorts, systemic exposure comparisons in cycle 1 (AUC) indicated that the addition of CHP to pola and obinutuzumab did not appear to substantially impact the PK of pola. The GMR for cycle 1 AUC comparisons (for DLBCL in Study GO29044 vs. DLBCL in Study GO27834) was 0.805 (90% CI 0.691–0.938) for acMMAE, and 0.907 (90% CI 0.629–1.31) for unconjugated MMAE, for pola + G-CHP compared with pola + obinutuzumab only (Table 2). These differences were within the PK variability of each analyte and could also be attributed to differences in patient characteristics, and, given the acceptable safety profiles of all treatment arms, were not considered clinically meaningful.

### Pola PK in treatment-naïve versus R/R NHL using a population PK approach

All of the treatment-naïve patients in the analysis were from the current study (GO29044), while R/R patients were pooled from several other studies for comparison. Based on the integrated acMMAE–MMAE population PK model using a pCC approach, patients who were treatment naïve had approximately 20% higher central *V*_ss_ for acMMAE compared with patients with R/R disease. As shown in Table 3, treatment-naïve patients also had lower acMMAE exposures (by 8% for AUC, and 15% for *C*_max_) and lower unconjugated MMAE exposures (by 26% for both AUC and *C*_max_) than patients with R/R disease (cycle 6), but these differences were small and within the CV%, so were not considered clinically relevant given lower unconjugated MMAE exposures would not adversely affect safety. As mentioned in the method section, as both differences in line of therapy and disease type were present between studies, the impact of the covariate of ‘treatment-naïve versus R/R’ status was indistinguishable from the impact of the combination with CHP versus without CHP.

**Table 3:** Population PK comparison of pola exposure (1.8 mg/kg q3w) at cycle 6 by treatment status (treatment-naïve vs. relapsed or refractory)

Pola analyte	Parameter	Geometric mean (% CV)		GMR (90% CI)
		R/R (*N* = 380)	Treatment-naïve (*N* = 80)	
acMMAE	AUC, ng day/mL	2950 (21)	2720 (15)	0.92 (0.89–0.96)
	*C*_max_, ng/mL	734 (15)	621 (17)	0.85 (0.82–0.88)
Unconjugated MMAE	AUC, ng day/mL	20.9 (50)	15.5 (42)	0.74 (0.68–0.80)
	*C*_max_, ng/mL	1.93 (46)	1.42 (37)	0.74 (0.68–0.80)

All treatment-naïve B-NHL patients (*N* = 80) were from the current Study GO29044, who received pola co-administered with CHP chemotherapy. Relapsed/refractory B-NHL patients (*N* = 380) were from Studies DCS4968g (NCT01290549), GO27834 (NCT01691898), and GO29365 (NCT02257567), which included patients who received either single-agent pola, pola with R/G, or pola with R/G and bendamustine

*acMMAE* antibody-conjugated MMAE, *AUC* area under the curve, *B-NHL* B-cell non-Hodgkin lymphoma, *CHP* cyclophosphamide, doxorubicin, and prednisone, *CI* confidence interval, *C*_*max*_ maximum concentration, *CV* coefficient of variation, *G* obinutuzumab, *GMR* geometric mean ratio, *MMAE*, monomethyl auristatin E, *PK* pharmacokinetic, *pola* polatuzumab vedotin, *q3w* every 3 weeks, *R* rituximab, *R/R* relapsed/refractory

### PK of rituximab in combination with pola + CHP, and the potential of pola to influence the PK of rituximab

After the first dose of rituximab 375 mg/m^2^, the geometric mean (CV%) serum *C*_max_ reached 165 (37.2) μg/mL (*N* = 24), increasing to 237 (18.3) μg/mL (*N* = 28) by cycle 4. The minimum concentration (*C*_min_) levels of rituximab following cycle 3 (cycle 4 pre-dose) in DLBCL patients in Study GO29044 had a GMR of 1.47 (90% CI 1.26–1.72) when compared with Study BO22334 (R-CHOP) in the absence of pola (Table 4). Differences observed with cross-study comparison of rituximab exposure are within the variability of rituximab observed in BO22334 (up to 111% CV). Furthermore, the impact of pola on rituximab has been formally assessed in a population PK analysis of rituximab (Roche/Genentech data on file). The model included rituximab PK data collected in the four studies of pola (DCS4968g, GO27834, GO29044, and GO29365) with or without co-administration of pola. Based on a covariate assessment with pola on rituximab PK, combination with pola was not a significant covariate of rituximab exposure.

**Table 4:** Assessment of 1.8 mg/kg of pola as a perpetrator of a PK drug–drug interaction with cyclophosphamide, doxorubicin, rituximab, and obinutuzumab as a victim based on descriptive statistics of exposure comparisons

DDI victim	Tx	Parameter	*N*	GO29044 (C1D1) Before pola dosing	*N*	GO29044 (C3D1) After pola dosing	GMR (90% CI)
Cyclophosphamide (µg/mL)	R-CHP	C_23h_	25	2.64 (56.2)	19	2.67 (74.8)	1.01 (0.737–1.38)
	G-CHP	C_23h_	14	3.00 (52.4)	14	2.83 (50.1)	0.943 (0.691–1.29)
Doxorubicin (ng/mL)	R-CHP	C_24h_	25	8.79 (29.1)	20	8.43 (25.8)	0.959 (0.838–1.10)
	G-CHP	C_24h_	12	9.44 (60.6)	14	8.94 (21.3)	0.947 (0.701–1.28)

DDI victim	Tx	Parameter	*n*	BO22334 R-CHOP	*n*	GO29044 Pola + R-CHP	GMR (90% CI)
Rituximab (µg/mL)	R-CHP	C4 pre-dose	189	45.0 (111)	28	66.3 (36.2)	1.47 (1.26–1.72)

DDI victim	Tx	Parameter	*n*	BO21003 G	*n*	GO29044 Pola + G-CHP	GMR (90% CI)
Obinutuzumab (µg/mL)	G-CHP	C2 pre-dose	74	378 (60.5)	15	266 (71.0)	0.703 (0.517–0.955)

All values are geometric mean (% geo CV), except for GMR. GO29044 = 1L DLBCL; BO21003 = R/R indolent B-cell NHL; and BO22334 = 1L FL

*1L* first line, *C* cycle, *C*_*23h*_ concentration 23 h after dosing with DDI victim, *C*_*24h*_ concentration 24 h after dosing with DDI victim, *CI* confidence interval, *CV* coefficient of variation, *D* day, *DDI* drug–drug interaction, *DLBCL* diffuse large B-cell lymphoma, *FL* follicular lymphoma, *G* obinutuzumab, *G-CHP* obinutuzumab, cyclophosphamide, doxorubicin, and prednisone, *GMR* geometric mean ratio, *NHL* non-Hodgkin lymphoma, *PK* pharmacokinetic, *pola* polatuzumab vedotin, *R-CHOP* rituximab, cyclophosphamide, doxorubicin, vincristine, and prednisone, *R-CHP* rituximab, cyclophosphamide, doxorubicin, and prednisone, *R/R* relapsed/refractory, *tx* treatment

### PK of obinutuzumab in combination with pola and CHP, and the potential of pola to influence the PK of obinutuzumab

Following the first intravenous (IV) infusion of obinutuzumab 1000 mg, the geometric mean (CV%) serum *C*_max_ was 346 (20.4) μg/mL (*N* = 11). Matching obinutuzumab dosing regimens for patients with DLBCL in Study GO29044 up to cycle 2 in patients with B-NHL in Study BO21003 allowed for a cross-study comparison of pola + G-CHP to single-agent obinutuzumab therapy based on cycle 2 pre-dose concentrations. The cycle 2 pre-dose concentration of obinutuzumab was lower when administered with pola + CHP (Study GO29044) versus administration as a monotherapy (Study BO21003); the GMR was 0.703 (90% CI 0.517–0.955), as shown in Table 4. This difference was well within the variability of obinutuzumab (60% CV in Study BO21003), and may reflect variability in body weight, gender, and tumor burden due to differences in patient populations between the studies (e.g., DLBCL in GO29044 vs. R/R indolent B-cell NHL in BO21003).

### PK of cyclophosphamide and doxorubicin in combination with pola, prednisone, rituximab or obinutuzumab, and the potential of pola to influence the PK of cyclophosphamide and doxorubicin

Cyclophosphamide and doxorubicin PK were measured in patients with DLBCL prior to and after the administration of pola. After IV infusion of the first dose of cyclophosphamide and before administration of pola, minimum plasma concentrations (*C*_min_) of cyclophosphamide had a GMR of 1.01 (90% CI 0.737–1.38) for R-CHP and 0.943 (90% CI 0.691–1.29) for G-CHP compared with subsequent concomitant dosing with pola by cycle 3. Similarly, after IV infusion of the first dose of doxorubicin and before administration of pola, *C*_min_ of doxorubicin had a GMR of 0.959 (90% CI 0.838–1.10) for R-CHP and 0.947 (90% CI 0.701–1.28) for G-CHP compared with subsequent concomitant dosing with pola by cycle 3. These findings suggest that there is no impact of pola as a perpetrator on cyclophosphamide or doxorubicin PK (Table 3). Minimal differences in cyclophosphamide or doxorubicin PK were observed between DLBCL patients receiving either pola + R-CHP or pola + G-CHP.

## Discussion

ADCs are currently being evaluated in many different oncologic diseases, as consolidation or maintenance therapy, as single agents and in combination with other therapies, and in first-line and R/R settings [36–38]. Given the broad clinical versatility of these agents, theoretical DDI risks with ADCs related to the conjugate and cytotoxic drug exist when given in combination with another TP/SMD [28, 39–43]. Furthermore, as both ADCs and chemotherapeutic agents have narrow therapeutic windows, it is imperative that the effects of conventional drugs on the ADC, as well as the effect of the ADC on conventional drugs, are assessed [44]. A limit of 6–8 cycles of pola 1.8 mg/kg has been determined to be effective reducing the risk of peripheral neuropathy (a known adverse event [AE] with pola as a single agent) [45].

The potential impact of rituximab and obinutuzumab on the PK of pola was explored using a pooled population PK model analysis across multiple studies by a pCC approach assuming all patients are R/R for treatment status (unpublished data on file). The presence of rituximab combination was identified as a significant covariate in the model, with a mild impact on steady-state (cycle 6) exposure of acMMAE (24% higher for AUC) and moderately lower impact on steady-state exposure of unconjugated MMAE (37% lower for AUC and 40% lower for *C*_max_), which were within the inter-individual variability of both analytes and not considered clinically meaningful based on exposure–response analysis (unpublished data on file). A similar magnitude of impact was observed for the obinutuzumab combination as demonstrated by similar exposures (< 5% difference) compared with the rituximab combination.

The results of our investigation show that pola-related analytes have generally similar plasma/serum PK when given in combination with rituximab- and obinutuzumab-containing therapies, and between patients with mixed subtypes of B-NHL or DLBCL, and between treatment-naïve versus R/R status. Based on the range of geometric means across treatment arms in study GO29044, acMMAE was characterized by a *V*_ss_ mostly restricted to the central compartment (57.3–95.6 mL/kg), including a low CL (12.7–18.2 mL/kg/day) and long *t*_1/2_ (4.75–5.45 days), which is consistent with prior knowledge [14, 15]. Moderately higher acMMAE concentrations were seen in subsequent cycles with q3w dosing relative to cycle 1, which indicated accumulation, potentially due to time-dependent decrease of clearance of acMMAE (unpublished data on file). Unconjugated MMAE maximum plasma concentrations were substantially lower than those for acMMAE, with an average unconjugated MMAE *C*_max_ and AUC of < 2% of corresponding acMMAE values. The delayed *t*_max_ of unconjugated MMAE suggests a formation rate-limited kinetics whereby the rate of elimination of unconjugated MMAE may be limited by its slow release from the antibody-drug conjugate. Unconjugated MMAE did not accumulate in plasma with the q3w regimen.

Comparisons of cycle 1 exposures of cyclophosphamide and doxorubicin (prior to pola administration) were similar to those in cycle 3 (after pola administration), suggesting that pola does not impact the PK of cyclophosphamide or doxorubicin. The subtle differences observed in acMMAE exposure between the dose-escalation and expansion phases for patients receiving pola + R-CHP may be related to the relatively small dose-escalation sample size, and/or disease heterogeneity across studies. Similarly, the minor trend of slightly lower acMMAE and lower/higher unconjugated MMAE exposures in the presence of CHP, observed with both population PK and NCA approaches, may be attributed to the treatment-naïve condition, CHP effect, and/or study/individual PK variability. Significant overlap in the distribution of pola exposure exists, with the magnitude of differences being within the inter-individual variability of each analyte in the current study. These observations are not unexpected given the moderate inter-individual variability in exposure seen across other ADCs [46, 47].

The overall safety profile of pola added to R/G-CHP, including serious AEs, grade 3–4 AEs and events of peripheral neuropathy [45], is manageable and largely similar to treatment with R/G-CHOP, with vincristine, in the absence of pola administration in similar patient populations [19, 48]. Incorporation of 1.8 mg/kg of polatuzumab vedotin did not alter the safety profile described previously with R/G-CHOP in patients with DLBCL. While the AE profile in the current study varied by treatment arm, with a slightly larger incidence of grade 3–4 neutropenia and thrombocytopenia within the G-CHP arm compared to the R-CHP arm [18], exposures of acMMAE and unconjugated MMAE in both arms were similar at the 1.8-mg/kg dose level of pola. It has been shown that acMMAE is the key analyte driving both the safety and efficacy of pola [45, 49], although unconjugated MMAE is also associated with safety. In the current study, exposures of acMMAE and unconjugated MMAE did not appear to account for the slight discrepancy between the incidence of common AEs between treatment arms. Lastly, the dose intensity and need for dose modifications (dose delay/reduction, or drug discontinuation) were in line with the disease population undergoing cytotoxic therapies, suggesting that the combination of pola with immunochemotherapy was tolerable [19]. The population PK analysis described here also confirmed no clinically relevant impact of CHP combination or treatment-naïve status on the PK of either acMMAE or unconjugated MMAE.

Taken together, this report provides new insights and increases our clinical experience and understanding of pola pharmacokinetics when administered in combination with R/G-CHP, including the evaluation of the potential for DDI with the combination. Based on within-study and cross-study comparisons, there was no evidence for clinically meaningful DDIs among study treatments. The PK of pola was well characterized and consistent with expectations from previous studies employing pola, R/G, and/or CHP. No clinically meaningful differences of pola PK due to line of therapy (treatment-naïve vs. R/R status) were observed. The findings herein in addition to the favorable benefit/risk profile support the development strategy of pola + R/G-CHP as a first-line therapy in patients with B-NHL.

## Electronic supplementary material

Below is the link to the electronic supplementary material.

Supplementary file1 (DOCX 15 kb)

## References

[1] Chao MP (2013). Treatment challenges in the management of relapsed or refractory non-Hodgkin’s lymphoma—novel and emerging therapies. Cancer Manag Res.

[2] Bron D, Aurer I, Andre MPE, Bonnet C, Caballero D, Falandry C, Kimby E, Soubeyran P, Zucca E, Bosly A, Coiffier B (2017). Unmet needs in the scientific approach to older patients with lymphoma. Haematologica.

[3] Polson AG, Yu SF, Elkins K, Zheng B, Clark S, Ingle GS, Slaga DS, Giere L, Du C, Tan C, Hongo JA, Gogineni A, Cole MJ, Vandlen R, Stephan JP, Young J, Chang W, Scales SJ, Ross S, Eaton D, Ebens A (2007). Antibody-drug conjugates targeted to CD79 for the treatment of non-Hodgkin lymphoma. Blood.

[4] Prichard M, Harris T, Williams ME, Densmore JJ (2009). Treatment strategies for relapsed and refractory aggressive non-Hodgkin’s lymphoma. Expert Opin Pharmacother.

[5] Chu YW, Polson A (2013). Antibody-drug conjugates for the treatment of B-cell non-Hodgkin’s lymphoma and leukemia. Future Oncol.

[6] Peters C, Brown S (2015). Antibody-drug conjugates as novel anti-cancer chemotherapeutics. Biosci Rep.

[7] Kamath AV, Iyer S (2015). Preclinical pharmacokinetic considerations for the development of antibody drug conjugates. Pharm Res.

[8] Lambert JM, Morris CQ (2017). Antibody-drug conjugates (ADCs) for personalized treatment of solid tumors: a review. Adv Ther.

[9] Beck A, Goetsch L, Dumontet C, Corvaia N (2017). Strategies and challenges for the next generation of antibody-drug conjugates. Nat Rev Drug Discov.

[10] Lambert JM, Berkenblit A (2018). Antibody-drug conjugates for cancer treatment. Annu Rev Med.

[11] Dornan D, Bennett F, Chen Y, Dennis M, Eaton D, Elkins K, French D, Go MA, Jack A, Junutula JR, Koeppen H, Lau J, McBride J, Rawstron A, Shi X, Yu N, Yu SF, Yue SF, Yue P, Zheng B, Ebens A, Polson AG (2009). Therapeutic potential of an anti-CD79b antibody-drug conjugate, anti-CD79b-vc-MMAE, for treatment of non-Hodgkin lymphoma. Blood.

[12] Genentech, Inc. (2019) POLIVY™ (polatuzumab vedotin-piiq) for injection, for intravenous use. https://www.accessdata.fda.gov/drugsatfda_docs/label/2019/761121s000lbl.pdf. Accessed 4 July 2019

[13] Palanca-Wessels MC, Czuczman M, Salles G, Assouline S, Sehn LH, Flinn I, Patel MR, Sangha R, Hagenbeek A, Advani R, Tilly H, Casanovas O, Press OW, Yalamanchili S, Kahn R, Dere RC, Lu D, Jones S, Jones C, Chu YW, Morschhauser F (2015). Safety and activity of the anti-CD79B antibody-drug conjugate polatuzumab vedotin in relapsed or refractory B-cell non-Hodgkin lymphoma and chronic lymphocytic leukemia: a phase 1 study. Lancet Oncol.

[14] Morschhauser F, Flinn I, Advani RH, Diefenbach CS, Kolibaba K, Press OW, Sehn LH, Chen AI, Salles G, Tilly H, Cheson BD, Assouline S, Dreyling M, Hagenbeek A, Zinzani PL, Yalamanchili S, Lu D, Jones C, Jones S, Chu YW, Sharman JP (2014). Updated results of a phase II randomized study (ROMULUS) of polatuzumab vedotin or pinatuzumab vedotin plus rituximab in patients with relapsed/refractory non-Hodgkin lymphoma. Blood.

[15] Herrera AF, Molina A (2018). Investigational antibody-drug conjugates for treatment of B-linear malignancies. Clin Lymphoma Myeloma Leuk.

[16] Wolska-Washer A, Robak P, Smolewski P, Robak T (2017). Emerging antibody-drug conjugates for treating lymphoid malignancies. Expert Opin Emerg Drugs.

[17] Mehta A, Forero-Torres A (2015). Development and integration of antibody-drug conjugate in non-Hodgkin lymphoma. Curr Oncol Rep.

[18] Morschhauser F, Flinn IW, Advani R, Sehn LH, Diefenbach C, Kolibaba K, Press OW, Salles G, Tilly H, Chen AI, Assouline S, Cheson BD, Dreyling M, Hagenbeek A, Zinzani PL, Jones S, Cheng J, Lu D, Penuel E, Hirata J, Wenger M, Chu YW, Sharman J (2019). Polatuzumab vedotin or pinatuzumab vedotin plus rituximab in patients with relapsed or refractory non-Hodgkin lymphoma: final results from a phase 2 randomised study (ROMULUS). Lancet Haematol.

[19] Tilly H, Morschhauser F, Bartlett NL, Mehta A, Salles G, Haioun C, Munoz J, Chen AI, Kolibaba K, Lu D, Yan M, Penuel E, Hirata J, Lee C, Sharman JP (2019). Polatuzumab vedotin in combination with immunochemotherapy in patients with previously untreated diffuse large B-cell lymphoma: an open-label, non-randomised, phase 1b–2 study. Lancet Oncol.

[20] Lucas AT, Price LSL, Schorzman AN, Storrie M, Piscitelli JA, Razo J, Zamboni WC (2018). Factors affecting the pharmacology of antibody-drug conjugates. Antibodies.

[21] Tolcher AW (2016). Antibody drug conjugates: lessons from 20 years of clinical experience. Ann Oncol.

[22] Perez HL, Cardarelli PM, Deshpande S, Gangwar S, Schroeder GM, Vite GD, Borzilleri RM (2014). Antibody-drug conjugates: current status and future directions. Drug Discov Today.

[23] Vezina HE, Cotreau M, Han TH, Gupta M (2017). Antibody-drug conjugates as cancer therapeutics: past, present, and future. J Clin Pharmacol.

[24] US Department of Health and Human Services, Food and Drug Administration, Center for Drug Evaluation and Research (2012). Guidance for industry, drug interaction studies-study design, data analysis, implications for dosing, and labeling recommendations.

[25] European Medicines Agency, Committee for Human Medicinal Products (2015) Guideline on the investigation of drug interactions. 21 June 2012. European Medicines Agency, London

[26] Kraynov E, Kamath AV, Walles M, Tarcsa E, Deslandes A, Iyer RA, Datta-Mannan A, Sriraman P, Bairlein M, Yang JJ, Barfield M, Xiao G, Escandon E, Wang W, Rock DA, Chemuturi NV, Moore DJ (2016). Current approaches for absorption, distribution, metabolism, and excretion characterization of antibody-drug conjugates: an industry white paper. Drug Metab Dispos.

[27] Han TH, Zhao B (2014). Absorption, distribution, metabolism, and excretion considerations for the development of antibody-drug conjugates. Drug Metab Dispos.

[28] Lu D, Sahasranaman S, Yi Z, Girish S (2013). Strategies to address drug interaction potential for antibody-drug conjugates in clinical development. Bioanalysis.

[29] Kaur S, Xu K, Saad OM, Dere RC, Carrasco-Triguero M (2013). Bioanalytical assay strategies for the development of antibody-drug conjugate biotherapeutics. Bioanalysis.

[30] Gorovits B, Alley SC, Billic S, Booth B, Kaur S, Oldfield P, Purushothama S, Rao C, Shord S, Siguenza P (2013). Bioanalysis of antibody-drug conjugates: American Association of Pharmaceutical Scientists Antibody-drug Conjugate Working Group position paper. Bioanalysis.

[31] US Food and Drug Administration 2018 Bioanalytical method validation. https://www.fda.gov/files/drugs/published/Bioanalytical-Method-Validation-Guidance-for-Industry.pdf. Accessed 16 Jan 2020

[32] US Food and Drug Administration 2015 Analytical procedures and method validation for drugs and biologics. https://www.fda.gov/files/drugs/published/Analytical-Procedures-and-Methods-Validation-for-Drugs-and-Biologics.pdf. Accessed 16 Jan 2020

[33] European Medicines Agency (2011) Guideline on bioanalytical method validation. https://www.ema.europa.eu/en/documents/scientific-guideline/guideline-bioanalytical-method-validation_en.pdf. Accessed 16 Jan 2020

[34] Lu D, Lu T, Gibiansky L, Li X, Li C, Agarwal P, Shemesh CS, Shi R, Dere RC, Hirata J, Miles D, Chanu P, Girish S, Jin JY (2019). Integrated two-analyte population pharmacokinetic model of polatuzumab vedotin in patients with non-Hodgkin lymphoma. CPT Pharmacomet Syst Pharmacol.

[35] Phillips T, Brunvand M, Chen A, Press O, Essell J, Chiappella A, Diefenbach C, Jones S, Hirata J, Flinn IW (2016). Polatuzumab vedotin combined with obinutuzumab for patients with relapsed or refractory non-Hodgkin lymphoma: preliminary safety and clinical activity of a Phase Ib/II study. Blood.

[36] Drake PM, Rabuka D (2017). Recent developments in ADC technology: preclinical studies signal future clinical trends. BioDrugs.

[37] Mukherjee A, Waters AK, Babic I, Nurmemmedov E, Glassy MC, Kesari S, Yenugonda VM (2019). Antibody drug conjugates: progress, pitfalls, and promises. Hum Antibodies.

[38] De Goeij BE, Lambert JM (2016). New developments for antibody–drug conjugate-based therapeutic approaches. Curr Opin Immunol.

[39] Mahmood I, Green MD (2007). Drug interaction studies of therapeutic proteins or monoclonal antibodies. J Clin Pharmacol.

[40] Prueksaritanont T, Chu X, Gibson C, Cui D, Yee KL, Ballard J, Cabalu T, Hochman J (2013). Drug–drug interaction studies: regulatory guidance and an industry perspective. AAPS J.

[41] Kenny JR, Liu MM, Chow AT, Earp JC, Evers R, Slatter JG, Wang DD, Zhang L, Zhou H (2013). Therapeutic protein drug–drug interactions: navigating the knowledge gaps-highlights from the 2012 AAPS NBC Roundtable and IQ Consortium/FDA Workshop. AAPS J.

[42] Kamath AV, Iyer S (2016). Challenges and advances in the assessment of the disposition of antibody-drug conjugates. Biopharm Drug Dispos.

[43] Chen Y, Samineni D, Mukadem S, Wong H, Shen BQ, Lu D, Girish S, Hop C, Jin JY, Li C (2015). Physiologically based pharmacokinetic modeling as a tool to predict drug interactions for antibody-drug conjugates. Clin Pharmacokinet.

[44] Olivier KJ, Hurvitz SA (2016). Antibody–drug conjugates fundamentals, drug development, and clinical outcomes to target cancer.

[45] Lu D, Gillespie WR, Girish S, Agarwal P, Li C, Hirata J, Chu YW, Kagedal M, Leon L, Maiya V, Jin JY (2017). Time-to-event analysis of polatuzumab vedotin-induced peripheral neuropathy to assist in the comparison of clinical dosing regimens. CPT Pharmacomet Syst Pharmacol.

[46] Deslandes A (2014). Comparative clinical pharmacokinetics of antibody–drug conjugates in first-in-human Phase 1 studies. MAbs.

[47] Malik P, Phipps C, Edginton A, Blay J (2017). Pharmacokinetic considerations for antibody-drug conjugates against cancer. Pharm Res.

[48] Vitolo U, Trneny M, Belada D, Burke JM, Carella AM, Chua N, Abrisqueta P, Demeter J, Flinn I, Hong X, Kim WS, Pinto A, Shi YK, Tatsumi Y, Oestergaard MZ, Wenger M, Fingerle-Rowson G, Catalani O, Nielsen T, Martelli M, Sehn LH (2017). Obinutuzumab or rituximab plus cyclophosphamide, doxorubicin, vincristine, and prednisone in previously untreated diffuse large B-cell lymphoma. J Clin Oncol.

[49] Lu D, Jin JY, Gibiansky L, Gillespie WR, Agarwal P, Jones C, Chu YW, Wenger MK, Hirata J, Li C, Girish S (2015). Exposure-response analysis to assist selection of dose and treatment duration for polatuzumab vedotin as a single agent or in combination with rituximab for the treatment of B-cell lymphoma. Blood.

