# Supplementary material for: Pharmacokinetics of polatuzumab vedotin in combination with R/G-CHP in patients with B-cell non-Hodgkin lymphoma
Source: Cancer Chemother Pharmacol. 2020 Mar 28;85(5):831–42. doi: 10.1007/s00280-020-04054-8 (PMC7188703; doi:10.1007/s00280-020-04054-8)
Supplement: Supplementary file 1 — Supplementary file1 (DOCX 15 kb) [file 280_2020_4054_MOESM1_ESM.docx]

Supplementary material

**Article title:** Pharmacokinetics of polatuzumab vedotin in combination with R/G-CHP in patients with B-cell non-Hodgkin lymphoma

**Journal:** Cancer Chemotherapy and Pharmacology

**Authors:** Colby S. Shemesh, Priya Agarwal, Tong Lu, Calvin Lee, Randall C. Dere , Xiaobin Li, Chunze Li, Jin Y. Jin, Sandhya Girish, Dale Miles, Dan Lu

**Corresponding authors:** Colby S. Shemesh and Dan Lu, Genentech Inc; email shemesh.colby@gene.com, lu.dan@gene.com

Online Resource 1 Treatment and pharmacokinetic sampling scheme by study phase and treatment group

| **Treatment** | **Dose** | ***N* (histology)** | **Analyte** | **Timepoints** |
| --- | --- | --- | --- | --- |
| ***Phase Ib dose escalation*** | | | | |
| Pola + R-CHP | Pola: 1.0, 1.4, or 1.8 mg/kg  Rituximab: 375 mg/m^2^  Cyclophosphamide: 750 mg/m^2^  Doxorubicin: 50 mg/m^2^  Prednisone: 100 mg | 3–6 (B-NHL) per pola dose level | acMMAE, unconjugated MMAE | C1D2 0 and 0.5 h, C1D8, C1D15, C2D2, C3D1, and C4D1 0 and 0.5 h, PT |
| Pola + G-CHP | Pola: 1.4 or 1.8 mg/kg  Obinutuzumab: 1000 mg  Cyclophosphamide: 750 mg/m^2^  Doxorubicin: 50 mg/m^2^  Prednisone: 100 mg | 3–6 (B-NHL) per pola dose level | acMMAE, unconjugated MMAE | C1D2 0 and 0.5 h, C1D8, C1D15, C2D2, and C4D1 0 and 0.5 h, PT |
|  |  |  | Obinutuzumab | C1D1 0 h, C2D1 and C4D1 0 h, PT |
| ***Phase II expansion*** | | | | |
| Pola + R-CHP | Pola: 1.8 mg/kg  Rituximab: 375 mg/m^2^  Cyclophosphamide: 750 mg/m^2^  Doxorubicin: 50 mg/m^2^  Prednisone: 100 mg | 40 (DLBCL) | acMMAE, unconjugated MMAE | C1D2 0 and 0.5 h, C1D8, C1D15, C2D2, C3D1, and C4D1 0 and 0.5 h, TC/ET + PT |
|  |  |  | Cyclophosphamide | C1D1 and C3D1 post-infusion, 3 and 23 h |
|  |  |  | Doxorubicin | C1D1 and C3D1 2 and 24 h |
|  |  |  | Rituximab | C1D1 0 and 0.5 h, C4D1 0 and 0.5 h |
| Pola + G-CHP | Pola: 1.8 mg/kg  Obinutuzumab: 1000 mg  Cyclophosphamide: 750 mg/m^2^  Doxorubicin: 50 mg/m^2^  Prednisone: 100 mg | 17 (DLBCL) | acMMAE, unconjugated MMAE | C1D2 0 and 0.5 h, C1D8, C1D15, C2D2, C3D1, and C4D1 0 and 0.5 h, TC/ET + PT |
|  |  |  | Cyclophosphamide | C1D1 and C3D1 post-infusion, 3 and 23 h |
|  |  |  | Doxorubicin | C1D1 and C3D1 2 and 24 h |
|  |  |  | Obinutuzumab | C1D1, C2D1, and C4D1 0 h and post-infusion, PT |

Patients received up to a total of six (or eight) cycles. Rituximab, cyclophosphamide, and doxorubicin were administered on D1 of each 21-day cycle. Obinutuzumab was administered on D1, D8, D15 in Cycle 1, then D1 of each subsequent 21-day cycle. Pola was administered on D2 of Cycles 1 and 2, followed by D1 of subsequent 21-day cycles

*acMMAE*antibody-conjugated MMAE, *B-NHL* B-cell non-Hodgkin lymphoma, *C*cycle, *D* day, *DLBCL* diffuse large B-cell lymphoma, *ET* early term, *G-CHP* obinutuzumab, cyclophosphamide, doxorubicin, and prednisone, *MMAE* monomethyl auristatin E, *pola* polatuzumab vedotin, *PT* post treatment, *R-CHP* rituximab, cyclophosphamide, doxorubicin, and prednisone, *TC* treatment completion
